# EGCG-mediated autophagy flux has a neuroprotection effect via a class III histone deacetylase in primary neuron cells

**DOI:** 10.18632/oncotarget.3832

**Published:** 2015-04-14

**Authors:** Ju-Hee Lee, Ji-Hong Moon, Sung-Wook Kim, Jae-Kyo Jeong, Uddin MD Nazim, You-Jin Lee, Jae-Won Seol, Sang-Youel Park

**Affiliations:** ^1^ Biosafety Research Institute, College of Veterinary Medicine, Chonbuk National University, Jeonju, Jeonbuk, South Korea; ^2^ Department of Bioactive Material Sciences and Research Center of Bioactive Materials, Chonbuk National University, Jeonju, Jeonbuk, South Korea

**Keywords:** EGCG, Sirt1, autophagy, prion, neurotoxicity

## Abstract

Prion diseases caused by aggregated misfolded prion protein (PrP) are transmissible neurodegenerative disorders that occur in both humans and animals. Epigallocatechin-3-gallate (EGCG) has preventive effects on prion disease; however, the mechanisms related to preventing prion diseases are unclear. We investigated whether EGCG, the main polyphenol in green tea, prevents neuron cell damage induced by the human prion protein. We also studied the neuroprotective mechanisms and proper signals mediated by EGCG. The results showed that EGCG protects the neuronal cells against human prion protein-induced damage through inhibiting Bax and cytochrome c translocation and autophagic pathways by increasing LC3-II and reducing and blocking p62 by using ATG5 small interfering (si) RNA and autophagy inhibitors. We further demonstrated that the neuroprotective effects of EGCG were exhibited by a class III histone deacetylase; sirt1 activation and the neuroprotective effects attenuated by sirt1 inactivation using sirt1 siRNA and sirtinol. We demonstrated that EGCG activated the autophagic pathways by inducing sirt1, and had protective effects against human prion protein-induced neuronal cell toxicity. These results suggest that EGCG may be a therapeutic agent for treatment of neurodegenerative disorders including prion diseases.

## INTRODUCTION

Transmissible spongiform encephalopathies or prion diseases are neurodegenerative disorders that include bovine spongiform encephalopathy in cattle, chronic wasting disease in elk and deer, endemic scrapie of sheep and goats, and Creutzfeldt-Jacob disease and Gerstmann-Straussler-Scheinker syndrome in humans [1, 2]. Human prion diseases can occur fortuitously or by infection caused by mutation of the cellular prion protein (PrPc) [3]. An important step in the disease pathophysiology is conversion of cellular PrPc to disease-associated misfolded conformers or PrPsc [4]. These PrPsc are mainly composed of prion infectivity and are detectable in damaged brain and lympho-reticular organs [5].

The amino acid residue 106-126 of the cellular prion protein exhibits PrPsc characteristics including neuronal toxicity. This leads to fibril accumulation and mitochondrial damage, formation of amyloid-like fibrils *in vitro*, and proteinase K resistance [6, 7]. PrP (106-126) expresses significant toxicity to cultured cells, including primary rat hippocampus neuron cells, primary retinal cells, and PC12 cells [8, 9]. Furthermore, PrP (106-126) induces the accumulation of cytosolic PrP, which leads to enriched PrP in cell membranes [10].

Epigallocatechin-3-gallate (EGCG) is a natural component of green tea representing approximately 43% of its total phenol content [11]. EGCG has attracted attention as a potential therapeutic target in different areas of study, including neuronal diseases [12, 13], cardiovascular risk factors [14, 15], cancer [16], obesity, and type 2 diabetes [17, 18]. EGCG has protective effects against neuronal damage in diseases such as Alzheimer's disease (AD) and Parkinson's disease by blocking the formation of reactive oxygen species (ROS) and through iron chelating and anti-apoptotic functions in neurons [19-21].

Autophagy is an intracellular lysosomal process in which a double membrane vesicle (autophagosome) forms and sequesters the intracellular organelles and part of the cytoplasm. The autophagosome then fuses with a lysosome to form the autolysosome, exposing the inner compartment to lysosomal hydrolases for bulk degradation [22]. The formation of the autophagosome is mediated by the Atg12-Atg5-Atg16 complex and microtubule-associated protein light chain 3 (LC3-I)-phospholipid conjugates (LC3-II) [23, 24]. P62/SQSTM1 is well known autophagy substrate; p62 is incorporated into autophagosomes by interacting with LC3 and is efficiently degraded by autophagy. Inhibiting autophagy results in rapid accumulation of cellular p62, whereas decreased p62 levels are associated with activating autophagy; thus, p62 is employed as an indicator of autophagy flux [25].

Silent information regulator two ortholog 1 (sirt1) is a member of the sirtuin family and is a nicotinamide adenine dinucleotide-dependent histone deacetylase [26, 27]. It is the human ortholog of the yeast sir2 protein and is most likely one of the key proteins regulating caloric restriction-mediated lifespan extension [28]. Sirt1 removes acetyl groups from a variety of substrates such as the tumor suppressor protein p53, members of the FOXO family (forkhead box factors regulated by insulin/Akt), and hairy and enhancer of split 1. It is also related to physiological functions such as metabolism, cell survival, and aging [29, 30]. Sirt1 has been linked to neurodegenerative diseases such as Huntington's disease, AD, and Parkinson's disease neuropathology [29, 31]. sirt1 stimulates peroxisome proliferator-activated receptor gamma coactivator (PGC-1α) signaling [32] and AMPK activity by modulating deacetylation, activating LKB1 [33], and controlling the interaction between MnSOD and FoxO3a [34]. All of these key target proteins activated by sirt1 are essential for attenuating mitochondrial damage and oxidative stress [32, 35-37]. Furthermore, sirt1 protects against neurodegenerative diseases such as prion diseases by regulating mitochondrial homeostasis [38].

In some reports, EGCG stimulates the activity of the sirt1 regulatory protein [39]. Under stabilizing conditions, deacetylation of sirt1 is enhanced by diverse polyphenols such as EGCG, epicatechingallate, and myricetin (3.19-fold) [40]. A previous report showed that EGCG is an antagonist that suppresses oxidative stress induced by 1-methyl-4-phenyl-pyridine (MPP^+^) by modulating the sirt1/PGC-1α signaling pathway in PC12 cells [41]. Protective mechanisms of EGCG against cell damage by MMP^+^ and Cd^2+^-induced mitochondrial damage and neurodegenerative diseases such as Parkinson's disease have been suggested [41, 42]. The role of EGCG as an activator of autophagy in various cells such as macrophages [43], vascular endothelial cells [44], and hepatocytes [45] has been reported. In many cells, EGCG has health-associated benefits by activating autophagy against various diseases, including cardiovascular disorders, cancer, and atherosclerosis disorders. Although some reports have demonstrated that EGCG induces autophagy [44] or the sirt1 pathway [41] respectively, EGCG controlling autophagy via sirt1 has not been reported. Thus, this present study focused on the induction of autophagy and activation of the sirt1 pathway by EGCG on PrP (106-126)-induced neurotoxicity and mitochondrial damage in SH-SY5Y cells. The data support the hypothesis that EGCG-mediated autophagy via sirt1 expression and activation may be a therapeutic implication for prion disease and mitochondrial damage caused by prions.

## RESULTS

### EGCG has neuroprotective effects against PrP (106-126)-induced neurotoxicity

**Figure 1:**
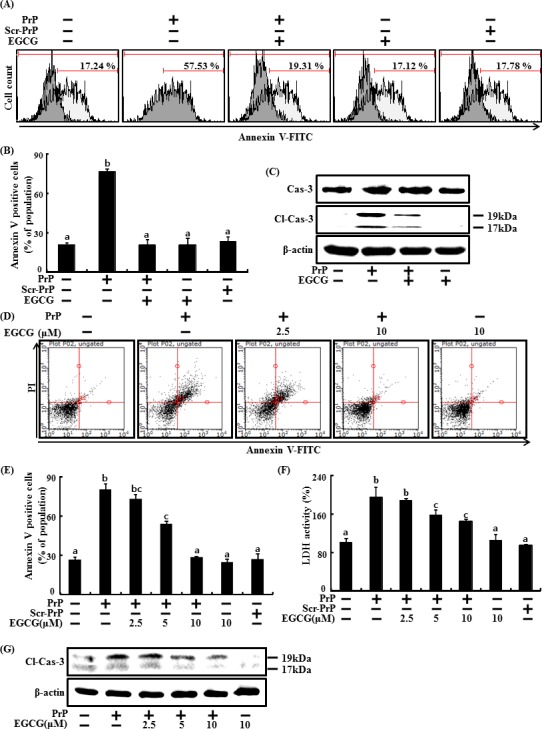
EGCG prevents neuronal cells from PrP (106-126)-induced cell death The primary neuron cells were pretreated with 10 μM of EGCG for 1 hr and then exposed to 50 μM PrP (106-126) or scrambled PrP (106-126) (scr-PrP) for 36 hr. Cell viability was measured by the Annexin V assay **A**, **B**. Western blot for cleaved caspases 3 protein was conducted with primary neuron cells. Beta-actin was used as the loading control **C**. SH-SY5Y cells were pretreated with 2.5, 5, or 10 μM of EGCG for 1 hr and then exposed to 50 μM PrP (106-126) or scrambled PrP (106-126) (scr-PrP) for 36 hr. Cell viability was measured by the Annexin V assay **D**, **E**. Lactate dehydrogenase (LDH) assay was used to quantify LDH released into the medium **F**. Western blot for cleaved caspases 3 protein was conducted with SH-SY5Y cells. Beta-actin was used as the loading control **G**. Bars indicate mean ± standard error (*n* = 4). The data were analyzed using ANOVA and Tukey multiple range tests (*P* < 0.01). Means sharing a common alphabetical symbol did not significantly differ. Cl-Cas-3, Cleaved caspase-3.

Many studies have shown that EGCG limits brain inflammation and reduces neuronal damage [19]. In this study, the neuroprotective effect of EGCG on PrP (106-126)-induced neuronal cell death (Figure 1) was confirmed. The primary neuron cells were pretreated with EGCG at concentration of 10 μM for 1 hr and then exposed to 50 μM PrP (106-126) for 36 hr. The primary neuron cells were measured for cell viability using Annexin V assay (Figure 1A, 1B). Without EGCG, only PrP (106-126)-treated cells exhibits cytotoxicity, and EGCG-treated cells appeared increased cell viability. To evaluate whether EGCG prevents apoptotic signaling, we confirmed the cleaved caspases 3 protein levels. Increased cleaved caspases 3 by treating PrP (106-126) was restored similarly to control group.

SH-SY5Y cells were pre-incubated with EGCG at concentrations of 1 and 10 μM for 1 hr and then exposed to 50 μM PrP (106-126) for 36 hr. When neuronal cells were treated with PrP (106-126) without EGCG, it resulted in neurotoxicity as measured by Annexin V (Figure 1D, 1E). Treatment with EGCG prevented PrP (106-126)-induced apoptosis in a dose-dependent manner. We also measured cell viability against scrambled PrP (106-126) as a control condition for PrP 106-126. As a result, cell death caused by scrambled PrP (106-126) did not occur. LDH release levels showed that EGCG inhibited PrP (106-126)-induced apoptosis in a dose-dependent manner but scrambled PrP did not lead to cell death (Figure 1F), consistent with previous results. Cleaved caspase 3 increased by PrP (106-126) was attenuated by EGCG treatment in a dose-dependent manner (Figure 1G).

### EGCG inhibits the PrP (106-126)-induced mitochondrial apoptotic pathway

**Figure 2:**
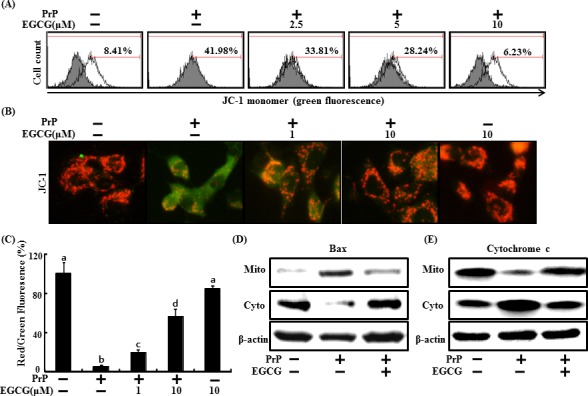
EGCG protects PrP (106-126)-induced mitochondrial damage SH-SY5Y cells were pretreated with 2.5, 5, or 10 μM of EGCG for 1 hr and then exposed to 50 μM PrP (106-126) for 36 hr. The treated cells were measured for JC-1 mono form (green) by flow cytometry. Percent values in the histogram represent the population of JC-1 monomeric cells. **A.** The treated cells were measured for JC-1 aggregates form (red) and mono form (green) by confocal microscopy analysis. Scale bar = 50 μm **B**. Separation of the cytosol and mitochondrial extracts was analyzed by Western blot using antibodies, to cytochrome c and Bax protein **D**, **E**. Bars indicate mean ± standard error (*n* = 4). The data were analyzed using ANOVA and Tukey multiple range tests (*P* < 0.01). Means sharing a common alphabetical symbol did not significantly differ.

Mitochondrial dysfunction is related to neuronal cell death caused by prions [46]. This study also examined whether an increase in Sirt1 activity causes prion-mediated mitochondrial impairment. Treatment of 50 μM PrP (106-126) increased green fluorescence indicating a lower MTP, whereas decreased PrP (106-126)-induced green fluorescence in cells pre-treated with EGCG (10 μM) (Figure 2A). These data were reconfirmed by measuring JC-1 using fluoroscopy (Figure 2B), PrP (106-126) treatment induced green fluorescence indicating a lower MTP, and PrP (106-126) treatment changed red fluorescence indicating a normal MTP following EGCG pretreatment (Figure 2B and 2C). In the same manner, PrP (106-126)-treated cells led to Bax translocation in the mitochondria, which increased cytochrome *c* release into the cytosol, whereas PrP (106-126)-induced Bax translocation and cytochrome *c* release decreased following EGCG treatment in SH-SY5Y cells. EGCG prevented PrP (106-126)-induced Bax translocation and cytochrome *c* release (Figure 2D and 2E). These data suggest that EGCG treatment inhibits the PrP (106-126)-mediated mitochondrial apoptotic pathway.

### EGCG inhibits the PrP (106-126)-induced apoptotic pathway by autophagy

We used autophagy as a survival strategy for prion-mediated neurotoxicity.

**Figure 3:**
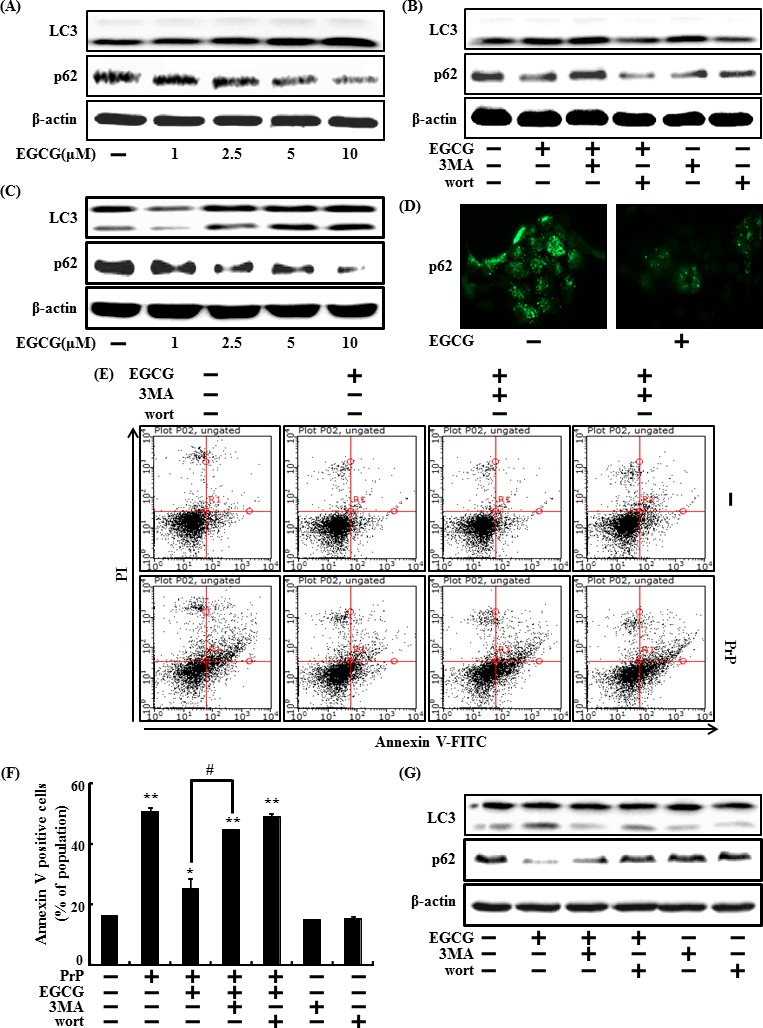
EGCG increases the induction of autophagy The primary neuron cells were treated with 1, 2.5, 5, 10 μM of EGCG for 30 h. Western blot for LC3-II, and p62 proteins was analyzed from primary neuron cells. Beta-actin was used as a loading control **A**. The cells were immunostained with p62 antibody (green) and observed in fluorescent view. Primary neuron cells were pretreated with 10 μM of EGCG in presence of autophagy inhibitor (3MA and wortmannin) for 30 h and western blot for LC3-II and p62 proteins was analyzed **B**. SH-SY5Y cells were treated with 2.5, 5, or 10 μM EGCG for 30 hr. Western blot for LC3-II, and p62 proteins was conducted with SH-SY5Y cells. Beta-actin was used as the loading control **C**. SH-SY5Y cells were analyzed by immunocytochemistry for p62 **D**. The cells were immunostained with p62 antibody (green) and observed in fluorescent view. SH-SY5Y cells were pretreated with 10 μM of EGCG in the presence of autophagy inhibitors (3MA or wortmannin) for 1 hr and then exposed to 50 μM PrP (106-126) for 36 h. Cell viability was measured by the Annexin V assay **E**, **F**. SH-SY5Y cells were treated as described in Figure 3A and Western blot for the LC3-II and p62 proteins was analyzed **G**. Bars indicate mean ± standard error (*n* = 4). *p < 0.05, **p < 0.01, significant differences between control and each treatment group, and #*p* < 0.01; significantly different when compared with PrP (106-126)-treated group.

First, we examined whether EGCG induces autophagy in primary neuron cells. LC3-II and p62 expression levels were detected using a specific antibody in a Western blot analysis. The primary neuron cells were treated with EGCG at concentrations of 1, 2.5, 5, or 10 μM for 24hr. EGCG increased LC3-II expression levels but decreased p62 levels (Figure 3A)

And also we confirmed the effects of EGCG in the SH-SY5Y cells. SH-SY5Y cells were treated with EGCG (1, 2.5, 5, or 10 μM, respectively) for 24 hr. EGCG increased LC3-II expression levels but decreased p62 levels (Figure 3C), and ICC also showed the reduction of p62 (Figure 3D). These data suggest that EGCG induced autophagy in SH-SY5Y cells.

We further tested to see whether inhibiting autophagy using autophagy inhibitors (3MA and wortmannin) could decrease the EGCG-induced neuroprotective effects against PrP (106-126). SH-SY5Y cells were pre-treated with autophagy inhibitors for 1 hr and then exposed to EGCG 10 μM in presence of PrP (106-126). As shown in Figure 3E and 3F, the neuroprotective effects of the EGCG treatment decreased following application of the autophagy inhibitors. And also, in protein levels in the primary neuron cells and SH-SY5Y cells, the autophagy inhibitors restored LC3- protein II expression levels and decreased p62 by EGCG treatment, compared to those in the control (Figure 3B and 3G).

**Figure 4:**
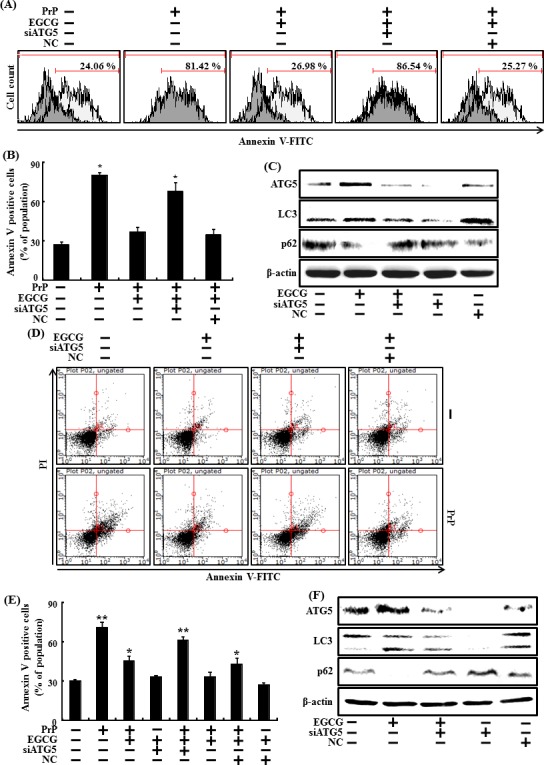
Inhibiting autophagy with ATG5 siRNA reduced the increase in autophagy caused by EGCG ATG5 small interfering RNA (siATG5) or negative control siRNA (NC) transfected primary neuron cells were incubated with 50 μM PrP (106-126) for 36 h in the presence of EGCG. Cell viability was measured by annexin V assay **A**, **B.** siATG5 or NC transfected primary neuron cells were incubated with EGCG (10 μM) for 30 h. Western blot for ATG5, LC3 and p62 proteins was analyzed from SH-SY5Y cells. β-actin was used as loading control **C.** siATG5 or NC transfected SH-SY5Y cells were incubated with 50 μM PrP (106-126) for 36 hr in the presence of EGCG. Cell viability was measured by Annexin V assay **D**, **E.** siATG5 or NC transfected SH-SY5Y cells were incubated with EGCG (10 μM) for 30 hr. Western blot for ATG5, LC3-II and p62 proteins was analyzed from SH-SY5Y cells. Beta-actin was used as the loading control **F.** Bars indicate mean ± standard error (n = 4). *p < 0.05, **p < 0.01, significant differences between control and each treatment group, and #*p* < 0.01; significantly different when compared with PrP (106-126)-treated group.

Knockdown of ATG5 using ATG5 small interfering RNA (ATG5 siRNA) decreased EGCG-induced autophagy (Figure 4C), as well as attenuated the neuroprotective effects caused by EGCG in both primary neuron cells (Figure 4A and 4B) and SH-SY5Y cells (Figure 4D and 4E)

### EGCG inhibits the PrP (106-126)-induced apoptotic pathway by sirt1

**Figure 5:**
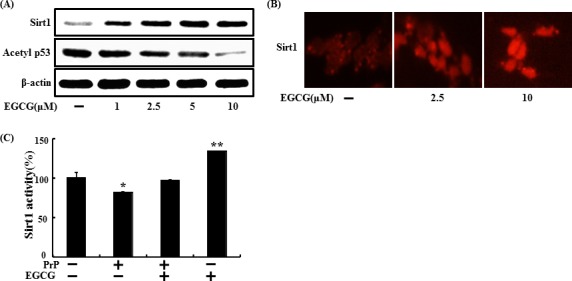
EGCG upregulates sirt1 expression and activation SH-SY5Y cells were treated with 2.5, 5, or 10 μM of EGCG for 30 hr. Western blot for sirt1 and acetyl-p53 proteins was analyzed from SH-SY5Y cells. Beta-actin was used as the loading control **A**. SH-SY5Y cells were analyzed by immunocytochemistry for sirt1 **B**. The cells were immunostained with sirt1 antibody (red) and observed in fluorescent view. SH-SY5Y cells were pretreated with 10 μM EGCG for 1 hr and then exposed to 50 μM PrP (106-126) for 36 hr. Sirt1 deacetylase activities in were analyzed in the nuclei of SH-SY5Y cells **C**. Bars indicate mean ± standard error (*n* = 4). *p < 0.05, **p < 0.01 significant differences between control and each treatment group.

sirt1 regulates key genes in neuronal survival [47] and stimulation of sirt1 is strongly affected by EGCG stability and metabolism [40]. To determine whether increased sirt1 was expressed, SH-SY5Y cells were treated with EGCG for 24 hr (Figure 5A). Sirt1 protein expression increased in a dose-dependent manner, and the result was reconfirmed by ICC (Figure 5B). Sirt1 binds with diverse transcription factors such as tumor suppressor p53, NF-κB p65, and FOXOs. Because p53 is an important regulator of apoptosis induced by prion fragments [48, 49], it was thought that these factors may be related to EGCG-induced neuroprotection against the prion protein. p53 is mainly used as a DNA damage marker in the neuroblastoma cell line [50, 51]. Additionally, deacetylation of p53 results in a decrease in apoptotic cell death in neurons [52, 53]. These studies demonstrate that sirt1 plays an important role in neuroprotection by deacetylating and inhibiting p53-induced and NFκB-induced inflammatory and apoptotic pathways. Sirt1 knockdown increased acetylated p53 level, leading to apoptosis in neurons. Hence, we examined whether EGCG contributed to deacetylation of p53 (Figure 5A). Acetylation of the p53 protein decreased in EGCG-treated cells. Consequently, EGCG up-regulated sirt1 activity, and treatment with PrP (106-126) resulted in sirt1 inactivation (Figure 5C). These results suggest that EGCG treatment is involved in both sirt1 expression and activation.

**Figure 6:**
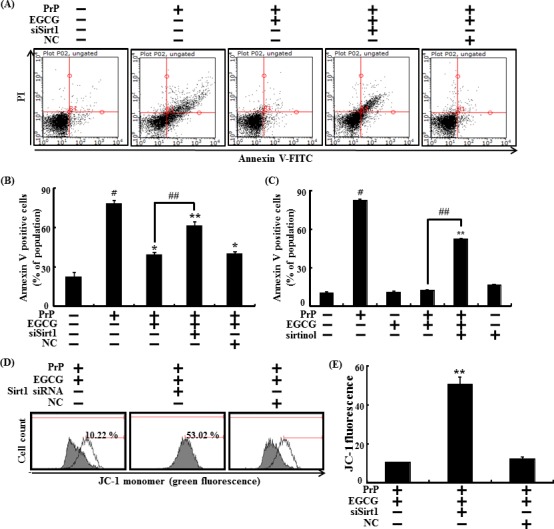
EGCG prevents neuronal cells from PrP (106-126)-induced cell death through the sirt1 pathway Sirt1 small interfering RNA (siSirt1) or negative control siRNA (NC) transfected SH-SY5Y cells were incubated with 50 μM PrP (106-126) for 36 hr in the presence of EGCG. Cell viability was measured by Annexin V assay **A**, **B**. SH-SY5Y cells were pretreated with sirtinol (10 μM) and EGCG (10 μM) for 1 h and then exposed to 50 μM PrP (106-126) for 36 hr. Cell viability was measured by Annexin V assay **C**. The cells were measured for JC-1 mono form (green) by flow cytometry. M1 represents the population of JC-1 monomeric cells **D**, **E**. Bars indicates mean ± standard error (*n* = 4). *p < 0.05, **p < 0.01, #*p* < 0.001 significant differences between control and each treatment group, and ##*p* < 0.01; significantly different when compared with PrP (106-126)-treated group.

Furthermore, sirt1 siRNA was used to knockdown sirt1 gene expression, to confirm that EGCG protects against prion-induced apoptosis by activating sirt1 and increasing sirt1 expression. We found that knockdown of sirt1 expression blocked the neuronal protective effects caused by EGCG, which inhibited neurotoxicity by PrP (106-126) (Figure 6A and 6B). No EGCG mediated-neuroprotective effects were observed when sirt1 activation was inhibited by sirtinol (sirt1 activation inhibitor) (Figure 6C). These results show that the protective effect on the MTP by activating sirt1 was inhibited by transfection with sirt1 siRNA. The SH-SY5Y cells pre-treated with EGCG 10 μM and exposed to 50 μM PrP (106-126) decreased PrP (106-126)-induced JC-1 monomer fluorescence intensity, whereas cells transfected with sirt1 siRNA increased JC-1 monomer fluorescence intensity (Figure 6D and 6E).

**Figure 7:**
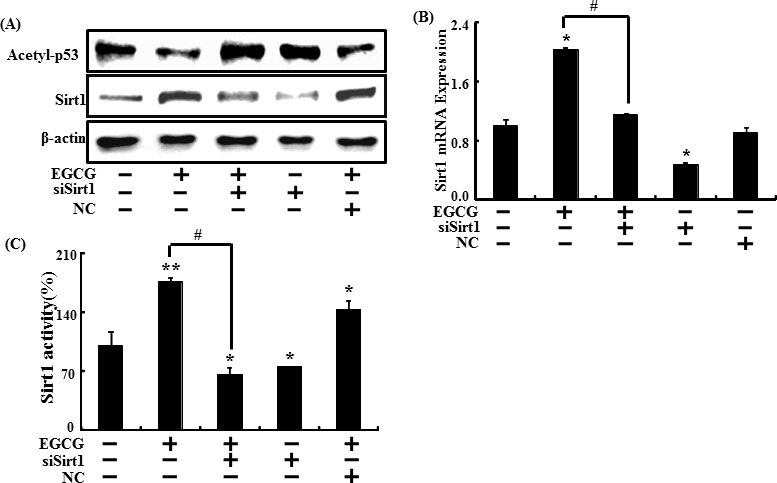
Inhibiting sirt1 decreased the sirt1 increase caused by EGCG Sirt1 small interfering RNA (siSirt1) or negative control siRNA (NC) transfected SH-SY5Y cells were incubated with EGCG (10 μM) for 30 hr. Western blot for sirt1 and acetyl-p53 proteins was conducted from SH-SY5Y cells. β-actin was used as the loading control **A**. Relative sirt1 mRNA expression levels were analyzed using quantitative real-time polymerase chain reaction. The indicated relative gene expression level shows expression levels that were normalized to β-actin expression as the standard **B**. siSirt1 or NC transfected SH-SY5Y cells were pre-incubated with EGCG (10 μM) for 1 hr and then exposed to PrP (106-126) for 30 hr. Sirt1 deacetylase activities were analyzed in SH-SY5Y cell nuclei. **C**. Bars indicate mean ± standard error (*n* = 4). *p < 0.05, **p < 0.01, significant differences between control and each treatment group, and #*p* < 0.01; significantly different when compared with PrP (106-126)-treated group.

We next investigated whether sirt1 knockdown inhibited sirt1 protein levels. SH-SY5Y cells transfected with sirt1 siRNA were treated with 10 μM EGCG. As a result, EGCG increased sirt1 protein levels, which were blocked by sirt1 siRNA. Sirt1 knockdown contributed to p53 deacetylation, and p53 acetylation protein levels decreased in EGCG-treated cells (Figure 7A).

Sirt1 expression was measured by analyzing mRNA expression using RT-PCR to investigate whether sirt1gene expression was blocked by sirt1 siRNA. Sirt1 mRNA level increased when SH-SY5Y cells were exposed to EGCG. However, sirt1 gene knockdown using sirt1 siRNA decreased sirt1 expression, compared to EGCG-treated cells (Figure 7B). In the same manner, transfecting sirt1 siRNA blocked increased sirt1 activity caused by EGCG treatment. Sirt1 activity did not change in the negative control (Figure 7C). Thus, we conclude that EGCG suppresses neuronal toxicity and mitochondrial damage by modulating both sirt1 activation and expression.

**Figure 8:**
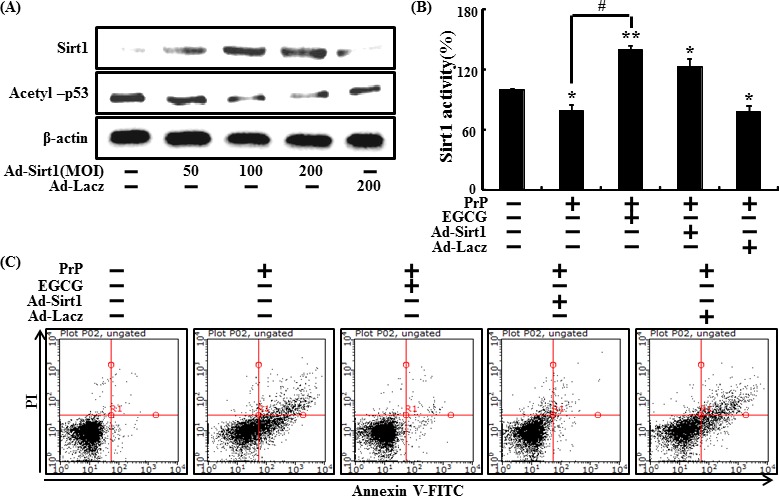
Overexpression of sirt1 and EGCG increased neuroprotective effects in SH-SY5Y cells SH-SY5Y cells were transfected by overexpressing adenovirus (Ad-Sirt1) or lacZ-bearing adenovirus (Ad-lacz). A Western blot for sirt1 and acetyl-p53 proteins was conducted from SH-SY5Y cells. Beta-actin was used as the loading control **A**. Ad-sirt1 or Ad-lacz transfected cells were incubated with or without EGCG and exposed to PrP (106-126) for 36 hr. Sirt1 deacetylase activities were analyzed in SH-SY5Y cell nuclei **B**. Cell viability was measured by the Annexin V assay **C**. Bars indicate mean ± standard error (*n* = 4). *p < 0.05, **p < 0.01, significant differences between control and each treatment group, and #*p* < 0.01; significantly different when compared with PrP(106-126)-treated group.

We also investigated whether sirt1 overexpression by adenoviral transfection up-regulated protein expression. Ad-Sirt1, transfected at a multiplicity of infection (MOI) of 10-100, gradually increased sirt1 protein expression with protein levels at 100 MOI (Figure 8A). Consequently, sirt1 overexpression and EGCG increased sirt1 activities (Figure 8B). We then tested if sirt1 overexpression increased the neuroprotective effect. EGCG-treated or Ad-sirt1 transfected cells increased the protective effects compared to Ad-Lacz transfected cells (Figure 8C). These results suggest that EGCG up-regulates sirt1 protein levels and activates sirt1.

### EGCG increase autophagy by activating sirt1

**Figure 9:**
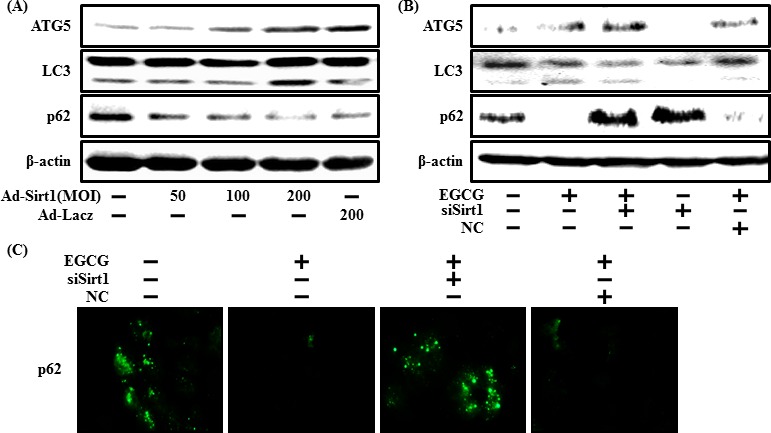
EGCG increases autophagy through the sirt1 pathway The SH-SY5Y cells were transfected by overexpressing adenovirus (Ad-Sirt1) or lacZ-bearing adenovirus (Ad-lacz). A Western blot of the LC3-II, ATG5 and p62 proteins was conducted in SH-SY5Y cells. Beta-actin was used as the loading control **A**. Sirt1 small interfering RNA (siSirt1) or negative control siRNA (NC) transfected SH-SY5Y cells were incubated with EGCG (10 μM) for 30 hr. A Western blot for the LC3-II, ATG5, and p62 proteins was conducted with SH-SY5Y cells. Beta-actin was used as the loading control **B**. SH-SY5Y cells were treated as described in Figure 3A, and then ICC for p62 was analyzed **C**.

Next, we checked whether increased autophagy caused by EGCG was related to activating sirt1. We first measured autophagy protein levels when sirt1 was overexpressed using the sirt1 adenovirus to investigate the relationship between autophagy and sirt1. We found that overexpression of sirt1 up-regulated LC3-II and decreased p62 (Figure 9A). We then examined whether sirt1 knockdown by sirt1 siRNA affected EGCG-induced autophagy (Figure 9B). Inhibiting sirt1 decreased ATG5 and LC3-II but and increased p62 levels. Consistent with these results, ICC also showed increased p62 expression when sirt1 was knocked down. We conclude that inhibiting sirt1 decreases EGCG-induced autophagy and EGCG prevents prion-mediated neurotoxicity by inducing autophagy and activating sirt1.

## DISCUSSION

This study demonstrated the possibility of attenuating prion protein-induced toxicity caused by EGCG, the major polyphenol in green tea, through autophagy activated by sirt1. We suggest that activating sirt1 with EGCG prevented mitochondrial damage related to prion-mediated toxicity. The biological effects of EGCG have already been demonstrated as it inhibits tumor growth of prostate, breast, liver, and lung cancers [4, 54, 55]. Studies have reported the potent effects of polyphenols against protein-misfolding diseases such as Alzheimer's, Parkinson's and Huntington's diseases.

In particular, EGCG leads to the formation of non-toxic amorphous aggregates through a disordered off-folding pathway [56] and modulates tau hyper-phosphorylation [57]. The protective effects of EGCG have also been investigated in different experimental models of neurodegenerative diseases [58-61]. One study showed that EGCG plays an important role with PrPc, to protect cells from stress, and to degrade non-native PrPc conformations [62]. However, it was unknown if EGCG prevents prion-mediated neurotoxicity and the pathway of its protective actions is unknown.

The main goal of this study was to identify the mechanism of how EGCG acts in prion disease by examining the neuronal effects of EGCG against PrP (106-126) toxicity in neuroblastoma SH-SY5Y cells. The active polyphenol EGCG shows various pharmacological effects such as antioxidant, antimutagenic, antiproliferative, and anticarcinogenic properties, as well as neuroprotective activities [63]. EGCG also play roles as a neuroprotective compound at multiple levels and can chelate metal ions and scavenge free radical species directly [64]. EGCG may cross the blood-brain barrier (BBB) [65] by a specific uptake mechanism for glucuronides *in vivo*. The degree of BBB penetration of EGCG depends on lipophilicity. Brain entry may also be modulated by specific efflux transporters expressed in the BBB such as P-glycoproteins.

EGCG interacts with various protein kinase and lipid kinase signaling cascades in the brain such as phosphatidylinositol-3 kinase (PtdIns 3K)/Akt, protein kinase C, and mitogen-activated protein kinase (MAPK) pathways. These interactions increase the number of neurons and strengthen connections between neurons via neurotropins such as brain derived neurotropic factor that support and maintain cognitive function. The chemical structures of the catechins, such as EGCG, contribute to their antioxidant properties. These compounds have a gallate moiety esterified site-specifically [42]. The potent free radical quenching activity of EGCG is affected by the presence of the gallate group [66]. Although we found protective effects of EGCG against apoptosis in neuroblastoma cells, many studies have revealed that EGCG induces non-apoptotic or apoptotic cell death in other cancer cells. Non-apoptotic cell death is stimulated by EGCG through autophagy and ROS [67, 68] and apoptosis occurs by activating caspases 3 and 9 [69]. Unlike other cancer cell lines, some studies have demonstrated that EGCG reduces apoptotic cell death in neuroblastoma cells [70, 71].

Autophagy is a complicated cellular process involved in cellular homeostasis and differentiation, as well as in tissue remodeling, aging, cancer, and other diseases [72]. During this process, targeted cytoplasmic constituents are isolated within autophagosomes, which are then fused with lysosomes for degradation or recycling [73]. We demonstrated that enhanced autophagy was caused by increased EGCG, due to increased expression of LC3-II and decreased expression of p62 in EGCG-treated SH-SY5Y cells, compared to control (Figure 3A). EGCG also prevented prion-mediated neuronal death (Figures 3 and 4). We further show the additional protective effect of EGCG as an autophagy inducer in SH-SY5Y cells. One study showed that EGCG may activate or inhibit various cellular signaling pathways such as NF-κB, sirt1, MAPK, heat shock proteins, and numerous additional regulatory molecules. We confirmed that EGCG activates the sirt1 pathway by blocking sirt1 expression using sirt1 siRNA and by overexpressing sirt1 using sirt1 adenovirus (Figures 5,6,7,8).

Our results suggest that regulating sirt1 activation may be important for preventing neurodegenerative conditions including prion diseases. Overexpression of sirt1 also protects against neurodegenerative diseases including Alzheimer's and Huntington's disease as well as amyotrophic lateral sclerosis [31, 74, 75]. A previous study demonstrated that activating sirt1 protects against prion-mediated neurotoxicity [76]. In addition, resveratrol, which is a sirt1 activator, protects against MPTP-mediated cell degeneration and mitochondrial-oxidative damage by activating the sirt1/PGC-1α pathways [77]. We showed here that prion-mediated neurotoxicity was protected by EGCG, as EGCG increased sirt1 protein expression and mRNA levels as well as sirt1 activities. Fewer neuroprotective effects were caused by sirt1 overexpression than by treatment with EGCG, indicating that although EGCG up-regulated sirt1 expression and activation, activating sirt1 has important effects beyond increased sirt1 expression.

We demonstrated that EGCG up-regulated autophagy via sirt1 as the main mechanism of neuroprotection against the prion peptide-induced apoptotic pathway. This result suggests that EGCG may have pivotal role as a therapeutic target for prion diseases. Because PrP (106-126) is not equal to PrPsc, we could only confirm PrPsc toxicity. However, the 106-126 sequence of the prion protein is a useful model for *in vitro* study of prion-induced cell death, as it results in rapid depolarization of mitochondrial membranes. Some studies have shown that PrP (106-126) induces apoptotic-mediated cell death *in vivo* in model retinal neurons treated with an intravitreous injection of PrP fragments. [78]. As we only conducted our experiments with EGCG in cells, and not animal models, the neuroprotective effects of EGCG have yet to be demonstrated *in vivo*. Therefore, we shall further study whether EGCG has a neuroprotective effect that occurs through sirt1 and autophagy pathway in PrP (106-126) mouse models to identify possible anti-prion drugs and potential therapeutic role in prion diseases.

## MATERIALS AND METHODS

### Cell culture

The primary neurons were isolated from individual 1 day old pups. Briefly, tissues were collected in Hanks Buffered Saline Solution without Mg2+ and Ca2+ (HBSS; Invitrogen-GIBCO, Grand Island, NY, USA), and digested in 0.6% trypsin with 2.78mM glucose (30min, 37°C, 5% CO2). Following trypsin neutralization with 1mg/ml soy trypsin inhibitor (Invitrogen, Carlsbad, CA, USA) containing 100U/ml DNAse I (Invitrogen, Carlsbad, CA, USA), cells were mechanically dissociated and diluted in HBSS containing Mg2+ and Ca2+. Isolated neuron cells were diluted in DMEM containing 25mM glucose (abbreviated in text as ‘glucose’ medium; Thermo scientific) supplemented with 10% FBS, and plated in flasks pre-coated with 50μg/ml poly D-lysine. The human neuroblastoma cell line SH-SY5Y was obtained from the American Type Culture Collection (ATCC, Rockville, MD, USA). Cells were cultured in Minimum Essential Medium (MEM, Hyclone Laboratories, Logan, UT, USA) containing 10% fetal bovine serum (Invitrogen-GIBCO, Grand Island, NY, USA) and gentamycin (0.1 mg/mL) in a humidified incubator maintained at 37°C and 5% CO_2_.

### PrP (106-126) treatment

Synthetic PrP (106-126) with the sequence Lys-Thr-Asn-Met-Lys-His-Met-Ala-Gly-Ala-Ala-Ala-Ala-Gly-Ala-Val-Val-Gly-Gly-Leu-Gly and scrambled PrP (106-126) (sequence, Asn-Gly-Ala-Lys-Ala-Leu-Met-Gly-Gly-His-Gly-Ala-Thr-Lys-Val-Met-Val-Gly-Ala-Ala-Ala) was synthesized by Peptron, Inc. (Seoul, Korea). The peptide was dissolved in sterile dimethyl sulfoxide at 10 mM and stored at −80°C.

### Annexin V assay

Apoptosis was assessed in detached cells using an Annexin V Assay kit (Santa Cruz Biotechnology, Santa Cruz, CA, USA), according to the manufacturer's protocol. Annexin V levels were determined by measuring fluorescence at 488 nm excitation and 525/30 emission using Guava EasyCyte HT System (Millipore, Bedford, MA, USA).

### Lactate dehydrogenase assay

Cytotoxicity was assessed in supernatants using a lactate dehydrogenase (LDH) cytotoxicity detection kit (Takara Bio, Inc., Tokyo, Japan) according to the manufacturer's protocol. LDH activity was determined by measuring absorbance at 490 nm, using a microplate reader (Spectra Max M2, Molecular Devices, Sunnyvale, CA, USA).

### Western blot analysis

After the SH-SY5Y cells were lysed in buffer (25 mM HEPES (4-(2-hydroxyethyl)-1-piperazineethanesulfonic acid), 100 mM NaCl, 1 mM EDTA, 5 mM MgCl_2_, 0.1 mM DTT, and a protease inhibitor mixture, at pH 7.4), the proteins were electrophoretically resolved by 10-15% sodium dodecyl sulfate polyacrylamide gel electrophoresis and transferred to a nitrocellulose membrane. Immunoreactivity was detected through sequential incubations with horseradish peroxidase-conjugated secondary antibodies and enhanced chemiluminescence reagents. The antibodies used for immunoblotting were LC3 (Novus Biologicals, Littleton, CO, USA), Bax (Santa Cruz Biotechnology, Santa Cruz, CA, USA), cytochrome *c* (BD BioScience, San Jose, CA, USA), autophagy related 5 (ATG5) (Abcam, Cambridge, MA, USA), Sirt1 (Santa Cruz Biotechnology, CA, USA), acetyl-p53 (Abcam, Cambridge, MA, USA) and β-actin (Sigma Aldrich, St. Louis, MO, USA). Images were examined using a Fusion FX7 imaging system (VilberLourmat, Torcy Z.I. Sud, France).

### Quantitative real-time polymerase chain reaction (qRT-PCR)

Total ribonucleic acid (RNA) was extracted from SH-SY5Y cells using the Easy-spin™ Total RNA Extraction kit (iNtRON Biotechnology, Seoul, Korea). The cDNA synthesis was carried out following the instructions of TaKaRa Prime Script TM 1st strand cDNA synthesis kit (TaKaRa Bio, Tokyo, Japan). For qRT-PCR, 1 μL of gene primers with SYBR Green (Bio-Rad Laboratories, Hercules, CA, USA) in 20 μL of reaction volume was applied. The primers were: SIRT1 forward, 5′TGCTCGCCTTGCTGTAGACTTC3′, reverse, 5′GGCTATGAATTTGTGACAGAGAGATGG3′; β-actin (as an internal control): forward, 5′GCAAGCAGGAGTATGACGAG3′, reverse, 5′CAAATAAAGCCATGCCAATC3′. All reactions with iTaq SYBR Green Supermix (Bio-Rad Laboratories, Hercules, CA, USA) were performed on the CFX96 real-time PCR detection system (Bio-Rad Laboratories, Hercules, CA, USA).

### Mitochondrial transmembrane potential assay

The change in mitochondrial transmembrane potential (MTP) was evaluated with a JC-1 cationic fluorescent indicator (Molecular Probes, Eugene, OR, USA) which aggregates in intact mitochondria (red fluorescence) indicating normal MTP and when it remains in monomeric form in the cytoplasm (green fluorescence) indicating low MTP. SH-SY5Y cells were incubated in MEM containing 10 μM JC-1 at 37°C for 15 min, washed with PBS, and then transferred to a clear 96-well plate. The JC-1 aggregate fluorescent emissions were measured at 583 nm with an excitation wavelength of 526 nm, whereas the JC-1 monomer fluorescence intensity was measured with excitation and emission at 525 and 530 nm, respectively, using a Guava EasyCyte HT System (Millipore, Billerica, MA, USA) or a microplate reader (Spectra Max M2, Molecular Devices). SH-SY5Y cells were cultured on coverslips in a 24-well plate, incubated in MEM containing 10 μM JC-1 at 37°C for 15 min, and then washed with PBS. Finally, the cells were mounted with Dako Fluorescent mounting medium and visualized via Nikon Eclipse 80i fluorescence microscopy (Nikon, Tokyo, Japan).

### Construction of recombinant adenoviruses

The Sirt1 over-expressing adenovirus (Ad-Sirt1) was provided by Professor Byung-Hyun Park of Chonbuk National University (Jeonju, Jeonbuk, South Korea). The lacZ-bearing adenovirus (Ad-lacZ) was used as a control. Recombinant adenoviruses were amplified in human embryonic kidney (HEK)-293 cells and purified using the Vivapure AdenoPACK kit (Sartorius AG, Göttingen, Germany) according to manufacturer's instructions.

### RNA interference

SH-SY5Y cells were transfected with ATG5 small interfering RNA (siRNA; oligoID HSS114104; Invitrogen, Carlsbad, CA, USA) or Sirt1 siRNA (oligoID VHS50608; Invitrogen) using Lipofectamine 2000 according to manufacturer's instructions. After a 48-hr culture, knockdown efficiency was measured at the protein level by immunoblot analysis. Nonspecific siRNA (oligoID 12935-300; Invitrogen) was used as the negative control.

### Sirt1 deacetylase activity assay

To measure cellular Sirt1 deacetylase activity, nuclear proteins were extracted from SH-SY5Y cells using a Nuclear/Cytosol Fractionation kit (BioVision, Milpitas, CA, USA). Sirt1 deacetylase activity was quantified following the protocols of the Sirt1 Fluorometric Assay kit (Sigma-Aldrich). Fluorescence intensities were measured with a microplate fluorometer (excitation wavelength = 360 nm, emission wavelength = 450 nm). The fluorescence intensities of Sirt1 deacetylase activity were normalized to the protein levels measured in the cell samples.

### Statistical evaluation

All data are expressed as mean ± standard deviation and were compared by Student's *t*-test, analysis of variance and Duncan's test using SAS statistical software ver. 9.1 (SAS Institute, Cary, NC, USA). Results were considered significant at *p < 0.05, **p < 0.001 and #*p* < 0.01.

## References

[1] Wickner RB, Edskes HK, Shewmaker F, Nakayashiki T (2007). Prions of fungi: inherited structures and biological roles. Nat Rev Microbiol.

[2] Tuite MF, Serio TR (2010). The prion hypothesis: from biological anomaly to basic regulatory mechanism. Nat Rev Mol Cell Biol.

[3] Collinge J (2001). Prion diseases of humans and animals: their causes and molecular basis. Annu Rev Neurosci.

[4] Lambert JD, Sang S, Hong J, Kwon SJ, Lee MJ, Ho CT, Yang CS (2006). Peracetylation as a means of enhancing *in vitro* bioactivity and bioavailability of epigallocatechin-3-gallate. Drug Metab Dispos.

[5] Krasemann S, Neumann M, Szalay B, Stocking C, Glatzel M (2013). Protease-sensitive prion species in neoplastic spleens of prion-infected mice with uncoupling of PrPSc and prion infectivity. J Gen Virol.

[6] Bergstrom AL, Cordes H, Zsurger N, Heegaard PM, Laursen H, Chabry J (2005). Amidation and structure relaxation abolish the neurotoxicity of the prion peptide PrP106-126 *in vivo* and *in vitro*. J Biol Chem.

[7] Selvaggini C, De Gioia L, Cantu L, Ghibaudi E, Diomede L, Passerini F, Forloni G, Bugiani O, Tagliavini F, Salmona M (1993). Molecular characteristics of a protease-resistant, amyloidogenic and neurotoxic peptide homologous to residues 106-126 of the prion protein. Biochemical and biophysical research communications.

[8] Gong J, Jellali A, Forster V, Mutterer J, Dubus E, Altrock WD, Sahel JA, Rendon A, Picaud S (2007). The toxicity of the PrP106-126 prion peptide on cultured photoreceptors correlates with the prion protein distribution in the mammalian and human retina. Am J Pathol.

[9] Forloni G, Angeretti N, Chiesa R, Monzani E, Salmona M, Bugiani O, Tagliavini F (1993). Neurotoxicity of a prion protein fragment. Nature.

[10] Hegde RS, Mastrianni JA, Scott MR, DeFea KA, Tremblay P, Torchia M, DeArmond SJ, Prusiner SB, Lingappa VR (1998). A transmembrane form of the prion protein in neurodegenerative disease. Science.

[11] Romeo L, Intrieri M, D'Agata V, Mangano NG, Oriani G, Ontario ML and, Scapagnini G (2009). The major green tea polyphenol, (−)-epigallocatechin-3-gallate, induces heme oxygenase in rat neurons and acts as an effective neuroprotective agent against oxidative stress. J Am Coll Nutr.

[12] Mandel S, Weinreb O, Amit T, Youdim MB (2004). Cell signaling pathways in the neuroprotective actions of the green tea polyphenol (−)-epigallocatechin-3-gallate: implications for neurodegenerative diseases. J Neurochem.

[13] Chao J, Lau WK, Huie MJ, Ho YS, Yu MS, Lai CS, Wang M, Yuen WH, Lam WH, Chan TH, Chang RC (2010). A pro-drug of the green tea polyphenol (−)-epigallocatechin-3-gallate (EGCG) prevents differentiated SH-SY5Y cells from toxicity induced by 6-hydroxydopamine. Neuroscience letters.

[14] Sano J, Inami S, Seimiya K, Ohba T, Sakai S, Takano T, Mizuno K (2004). Effects of green tea intake on the development of coronary artery disease. Circ J.

[15] Wolfram S (2007). Effects of green tea and EGCG on cardiovascular and metabolic health. J Am Coll Nutr.

[16] Moyers SB, Kumar NB (2004). Green tea polyphenols and cancer chemoprevention: multiple mechanisms and endpoints for phase II trials. Nutr Rev.

[17] Boschmann M, Thielecke F (2007). The effects of epigallocatechin-3-gallate on thermogenesis and fat oxidation in obese men: a pilot study. J Am Coll Nutr.

[18] Potenza MA, Marasciulo FL, Tarquinio M, Tiravanti E, Colantuono G, Federici A, Kim JA, Quon MJ, Montagnani M (2007). EGCG, a green tea polyphenol, improves endothelial function and insulin sensitivity, reduces blood pressure, and protects against myocardial I/R injury in SHR. Am J Physiol Endocrinol Metab.

[19] Aktas O, Prozorovski T, Smorodchenko A, Savaskan NE, Lauster R, Kloetzel PM, Infante-Duarte C, Brocke S, Zipp F (2004). Green tea epigallocatechin-3-gallate mediates T cellular NF-kappa B inhibition and exerts neuroprotection in autoimmune encephalomyelitis. J Immunol.

[20] Schroeder EK, Kelsey NA, Doyle J, Breed E, Bouchard RJ, Loucks FA, Harbison RA, Linseman DA (2009). Green tea epigallocatechin 3-gallate accumulates in mitochondria and displays a selective antiapoptotic effect against inducers of mitochondrial oxidative stress in neurons. Antioxidants & redox signaling.

[21] Hechler D, Nitsch R, Hendrix S (2006). Green-fluorescent-protein-expressing mice as models for the study of axonal growth and regeneration *in vitro*. Brain Res Rev.

[22] Levine B, Klionsky DJ (2004). Development by self-digestion: molecular mechanisms and biological functions of autophagy. Developmental cell.

[23] Kabeya Y, Mizushima N, Ueno T, Yamamoto A, Kirisako T, Noda T, Kominami E, Ohsumi Y, Yoshimori T (2000). LC3, a mammalian homologue of yeast Apg8p, is localized in autophagosome membranes after processing. The EMBO journal.

[24] Tanida I, Minematsu-Ikeguchi N, Ueno T, Kominami E (2005). Lysosomal turnover, but not a cellular level, of endogenous LC3 is a marker for autophagy. Autophagy.

[25] Mizushima N, Yoshimori T, Levine B (2010). Methods in mammalian autophagy research. Cell.

[26] Michan S, Sinclair D (2007). Sirtuins in mammals: insights into their biological function. Biochem J.

[27] Pillarisetti S (2008). A review of Sirt1 and Sirt1 modulators in cardiovascular and metabolic diseases. Recent Pat Cardiovasc Drug Discov.

[28] Guarente L (2005). Calorie restriction and SIR2 genes--towards a mechanism. Mech Ageing Dev.

[29] Haigis MC, Guarente LP (2006). Mammalian sirtuins--emerging roles in physiology, aging, and calorie restriction. Genes Dev.

[30] Yamamoto H, Schoonjans K, Auwerx J (2007). Sirtuin functions in health and disease. Mol Endocrinol.

[31] Kim D, Nguyen MD, Dobbin MM, Fischer A, Sananbenesi F, Rodgers JT, Delalle I, Baur JA, Sui G, Armour SM, Puigserver P, Sinclair DA, Tsai LH (2007). SIRT1 deacetylase protects against neurodegeneration in models for Alzheimer's disease and amyotrophic lateral sclerosis. EMBO J.

[32] Lagouge M, Argmann C, Gerhart-Hines Z, Meziane H, Lerin C, Daussin F, Messadeq N, Milne J, Lambert P, Elliott P, Geny B, Laakso M, Puigserver P, Auwerx J (2006). Resveratrol improves mitochondrial function and protects against metabolic disease by activating SIRT1 and PGC-1alpha. Cell.

[33] Lan F, Cacicedo JM, Ruderman N, Ido Y (2008). SIRT1 modulation of the acetylation status, cytosolic localization, and activity of LKB1. Possible role in AMP-activated protein kinase activation. J Biol Chem.

[34] Tanno M, Kuno A, Yano T, Miura T, Hisahara S, Ishikawa S, Shimamoto K, Horio Y (2010). Induction of manganese superoxide dismutase by nuclear translocation and activation of SIRT1 promotes cell survival in chronic heart failure. J Biol Chem.

[35] Danz ED, Skramsted J, Henry N, Bennett JA, Keller RS (2009). Resveratrol prevents doxorubicin cardiotoxicity through mitochondrial stabilization and the Sirt1 pathway. Free Radic Biol Med.

[36] Dasgupta B, Milbrandt J (2007). Resveratrol stimulates AMP kinase activity in neurons. Proc Natl Acad Sci U S A.

[37] Ding DF, You N, Wu XM, Xu JR, Hu AP, Ye XL, Zhu Q, Jiang XQ, Miao H, Liu C, Lu YB (2010). Resveratrol attenuates renal hypertrophy in early-stage diabetes by activating AMPK. Am J Nephrol.

[38] Jeong JK, Moon MH, Lee YJ, Seol JW, Park SY (2013). Autophagy induced by the class III histone deacetylase Sirt1 prevents prion peptide neurotoxicity. Neurobiol Aging.

[39] Queen BL, Tollefsbol TO (2010). Polyphenols and aging. Curr Aging Sci.

[40] de Boer VC, de Goffau MC, Arts IC, Hollman PC, Keijer J (2006). SIRT1 stimulation by polyphenols is affected by their stability and metabolism. Mech Ageing Dev.

[41] Ye Q, Ye L, Xu X, Huang B, Zhang X, Zhu Y, Chen X (2012). Epigallocatechin-3-gallate suppresses 1-methyl-4-phenyl-pyridine-induced oxidative stress in PC12 cells via the SIRT1/PGC-1alpha signaling pathway. BMC Complement Altern Med.

[42] Abib RT, Peres KC, Barbosa AM, Peres TV, Bernardes A, Zimmermann LM, Quincozes-Santos A, Fiedler HD, Leal RB, Farina M, Gottfried C (2011). Epigallocatechin-3-gallate protects rat brain mitochondria against cadmium-induced damage. Food Chem Toxicol.

[43] Li W, Zhu S, Li J, Assa A, Jundoria A, Xu J, Fan S, Eissa NT, Tracey KJ, Sama AE, Wang H (2011). EGCG stimulates autophagy and reduces cytoplasmic HMGB1 levels in endotoxin-stimulated macrophages. Biochemical pharmacology.

[44] Kim HS, Montana V, Jang HJ, Parpura V, Kim JA (2013). Epigallocatechin gallate (EGCG) stimulates autophagy in vascular endothelial cells: a potential role for reducing lipid accumulation. The Journal of biological chemistry.

[45] Zhang Y, Yang ND, Zhou F, Shen T, Duan T, Zhou J, Shi Y, Zhu XQ, Shen HM (2012). (−)-Epigallocatechin-3-gallate induces non-apoptotic cell death in human cancer cells via ROS-mediated lysosomal membrane permeabilization. PloS one.

[46] O'Donovan CN, Tobin D, Cotter TG (2001). Prion protein fragment PrP-(106-126) induces apoptosis via mitochondrial disruption in human neuronal SH-SY5Y cells. The Journal of biological chemistry.

[47] Gomes AP, Duarte FV, Nunes P, Hubbard BP, Teodoro JS, Varela AT, Jones JG, Sinclair DA, Palmeira CM, Rolo AP (2012). Berberine protects against high fat diet-induced dysfunction in muscle mitochondria by inducing SIRT1-dependent mitochondrial biogenesis. Biochim Biophys Acta.

[48] Bai Y, Li Q, Yang J, Zhou X, Yin X, Zhao D (2008). p75(NTR) activation of NF-kappaB is involved in PrP106-126-induced apoptosis in mouse neuroblastoma cells. Neurosci Res.

[49] Forloni G, Bugiani O, Tagliavini F, Salmona M (1996). Apoptosis-mediated neurotoxicity induced by beta-amyloid and PrP fragments. Mol Chem Neuropathol.

[50] Wang Y, Musich PR, Serrano MA, Zou Y, Zhang J, Zhu MY (2014). Effects of DSP4 on the Noradrenergic Phenotypes and Its Potential Molecular Mechanisms in SH-SY5Y Cells. Neurotox Res.

[51] Zhan Q, Tsai S, Lu Y, Wang C, Kwan Y, Ngai S (2013). RuvBL2 is involved in histone deacetylase inhibitor PCI-24781-induced cell death in SK-N-DZ neuroblastoma cells. PloS one.

[52] Hasegawa K, Yoshikawa K (2008). Necdin regulates p53 acetylation via Sirtuin1 to modulate DNA damage response in cortical neurons. The Journal of neuroscience: the official journal of the Society for Neuroscience.

[53] Hernandez-Jimenez M, Hurtado O, Cuartero MI, Ballesteros I, Moraga A, Pradillo JM, McBurney MW, Lizasoain I, Moro MA (2013). Silent information regulator 1 protects the brain against cerebral ischemic damage. Stroke; a journal of cerebral circulation.

[54] Lam WH, Kazi A, Kuhn DJ, Chow LM, Chan AS, Dou QP, Chan TH (2004). A potential prodrug for a green tea polyphenol proteasome inhibitor: evaluation of the peracetate ester of (−)-epigallocatechin gallate [(−)-EGCG]. Bioorg Med Chem.

[55] Lee SC, Chan WK, Lee TW, Lam WH, Wang X, Chan TH, Wong YC (2008). Effect of a prodrug of the green tea polyphenol (−)-epigallocatechin-3-gallate on the growth of androgen-independent prostate cancer *in vivo*. Nutr Cancer.

[56] Hudson SA, Ecroyd H, Dehle FC, Musgrave IF, Carver JA (2009). (−)-epigallocatechin-3-gallate (EGCG) maintains kappa-casein in its pre-fibrillar state without redirecting its aggregation pathway. Journal of molecular biology.

[57] Rezai-Zadeh K, Arendash GW, Hou H, Fernandez F, Jensen M, Runfeldt M, Shytle RD, Tan J (2008). Green tea epigallocatechin-3-gallate (EGCG) reduces beta-amyloid mediated cognitive impairment and modulates tau pathology in Alzheimer transgenic mice. Brain research.

[58] Chung WG, Miranda CL, Maier CS (2007). Epigallocatechin gallate (EGCG) potentiates the cytotoxicity of rotenone in neuroblastoma SH-SY5Y cells. Brain Res.

[59] Levites Y, Weinreb O, Maor G, Youdim MB, Mandel S (2001). Green tea polyphenol (−)-epigallocatechin-3-gallate prevents N-methyl-4-phenyl-1,2,3,6-tetrahydropyridine-induced dopaminergic neurodegeneration. J Neurochem.

[60] Levites Y, Youdim MB, Maor G, Mandel S (2002). Attenuation of 6-hydroxydopamine (6-OHDA)-induced nuclear factor-kappaB (NF-kappaB) activation and cell death by tea extracts in neuronal cultures. Biochem Pharmacol.

[61] Wang L, Xu S, Xu X, Chan P (2009). (−)-Epigallocatechin-3-Gallate protects SH-SY5Y cells against 6-OHDA-induced cell death through STAT3 activation. J Alzheimers Dis.

[62] Rambold AS, Miesbauer M, Olschewski D, Seidel R, Riemer C, Smale L, Brumm L, Levy M, Gazit E, Oesterhelt D, Baier M, Becker CF, Engelhard M, Winklhofer KF, Tatzelt J (2008). Green tea extracts interfere with the stress-protective activity of PrP and the formation of PrP. Journal of neurochemistry.

[63] Lee SR, Kim SP, Kim JE (2000). Protective effect of topiramate against hippocampal neuronal damage after global ischemia in the gerbils. Neurosci Lett.

[64] Perron NR, Brumaghim JL (2009). A review of the antioxidant mechanisms of polyphenol compounds related to iron binding. Cell Biochem Biophys.

[65] Lin LC, Wang MN, Tseng TY, Sung JS, Tsai TH (2007). Pharmacokinetics of (−)-epigallocatechin-3-gallate in conscious and freely moving rats and its brain regional distribution. Journal of agricultural and food chemistry.

[66] Devika PT, Stanely Mainzen Prince P (2008). (−)Epigallocatechin-gallate (EGCG) prevents mitochondrial damage in isoproterenol-induced cardiac toxicity in albino Wistar rats: a transmission electron microscopic and *in vitro* study. Pharmacol Res.

[67] Tao L, Forester SC, Lambert JD (2014). The role of the mitochondrial oxidative stress in the cytotoxic effects of the green tea catechin, (−)-epigallocatechin-3-gallate, in oral cells. Mol Nutr Food Res.

[68] Satoh M, Takemura Y, Hamada H, Sekido Y, Kubota S (2013). EGCG induces human mesothelioma cell death by inducing reactive oxygen species and autophagy. Cancer cell international.

[69] Gao Y, Li W, Jia L, Li B, Chen YC, Tu Y (2013). Enhancement of (−)-epigallocatechin-3-gallate and theaflavin-3-3′-digallate induced apoptosis by ascorbic acid in human lung adenocarcinoma SPC-A-1 cells and esophageal carcinoma Eca-109 cells via MAPK pathways. Biochemical and biophysical research communications.

[70] Tai KK, Truong DD (2010). (−)-Epigallocatechin-3-gallate (EGCG), a green tea polyphenol, reduces dichlorodiphenyl-trichloroethane (DDT)-induced cell death in dopaminergic SHSY-5Y cells. Neuroscience letters.

[71] Levites Y, Amit T, Youdim MB, Mandel S (2002). Involvement of protein kinase C activation and cell survival/cell cycle genes in green tea polyphenol (−)-epigallocatechin 3-gallate neuroprotective action. The Journal of biological chemistry.

[72] Yu Y, Shiou SR, Guo Y, Lu L, Westerhoff M, Sun J, Petrof EO, Claud EC (2013). Erythropoietin protects epithelial cells from excessive autophagy and apoptosis in experimental neonatal necrotizing enterocolitis. PloS one.

[73] Cuervo AM, Bergamini E, Brunk UT, Droge W, Ffrench M, Terman A (2005). Autophagy and aging: the importance of maintaining “clean” cells. Autophagy.

[74] Parker JA, Arango M, Abderrahmane S, Lambert E, Tourette C, Catoire H, Neri C (2005). Resveratrol rescues mutant polyglutamine cytotoxicity in nematode and mammalian neurons. Nat Genet.

[75] Qin W, Yang T, Ho L, Zhao Z, Wang J, Chen L, Zhao W, Thiyagarajan M, MacGrogan D, Rodgers JT, Puigserver P, Sadoshima J, Deng H, Pedrini S, Gandy S, Sauve AA (2006). Neuronal SIRT1 activation as a novel mechanism underlying the prevention of Alzheimer disease amyloid neuropathology by calorie restriction. J Biol Chem.

[76] Seo JS, Moon MH, Jeong JK, Seol JW, Lee YJ, Park BH, Park SY (2012). SIRT1, a histone deacetylase, regulates prion protein-induced neuronal cell death. Neurobiology of aging.

[77] Mudo G, Makela J, Di Liberto V, Tselykh TV, Olivieri M, Piepponen P, Eriksson O, Malkia A, Bonomo A, Kairisalo M, Aguirre JA, Korhonen L, Belluardo N, Lindholm D (2012). Transgenic expression and activation of PGC-1alpha protect dopaminergic neurons in the MPTP mouse model of Parkinson's disease. Cell Mol Life Sci.

[78] Ettaiche M, Pichot R, Vincent JP, Chabry J (2000). *In vivo* cytotoxicity of the prion protein fragment 106-126. The Journal of biological chemistry.

